# The miRNA Profile of Inflammatory Colorectal Tumors Identify TGF-β as a Companion Target for Checkpoint Blockade Immunotherapy

**DOI:** 10.3389/fcell.2021.754507

**Published:** 2021-10-14

**Authors:** Bjarne Bartlett, Zitong Gao, Monique Schukking, Mark Menor, Vedbar S. Khadka, Muller Fabbri, Peiwen Fei, Youping Deng

**Affiliations:** ^1^Bioinformatics Core, Department of Quantitative Health Sciences, University of Hawaii, Honolulu, HI, United States; ^2^Department of Molecular Biosciences and Bioengineering, University of Hawai‘i at Mānoa, Honolulu, HI, United States; ^3^Cancer Biology Program, University of Hawai‘i Cancer Center, Honolulu, HI, United States

**Keywords:** PD-L1, immunotherapy, checkpoint blockade, MSI, mutation burden

## Abstract

Extrinsic factors such as expression of PD-L1 (programmed dealth-ligand 1) in the tumor microenvironment (TME) have been shown to correlate with responses to checkpoint blockade therapy. More recently two intrinsic factors related to tumor genetics, microsatellite instability (MSI), and tumor mutation burden (TMB), have been linked to high response rates to checkpoint blockade drugs. These response rates led to the first tissue-agnostic approval of any cancer therapy by the FDA for the treatment of metastatic, MSI-H tumors with anti-PD-1 immunotherapy. But there are still very few studies focusing on the association of miRNAs with immune therapy through checkpoint inhibitors. Our team sought to explore the biology of such tumors further and suggest potential companion therapeutics to current checkpoint inhibitors. Analysis by Pearson Correlation revealed 41 total miRNAs correlated with mutation burden, 62 miRNAs correlated with MSI, and 17 miRNAs correlated with PD-L1 expression. Three miRNAs were correlated with all three of these tumor features as well as M1 macrophage polarization. No miRNAs in any group were associated with overall survival. TGF-β was predicted to be influenced by these three miRNAs (*p* = 0.008). Exploring miRNA targets as companions to treatment by immune checkpoint blockade revealed three potential miRNA targets predicted to impact TGF-β. M1 macrophage polarization state was also associated with tumors predicted to respond to therapy by immune checkpoint blockade.

## Introduction

Despite therapeutic advances and declining mortality since 1990, an estimated 50,630 patients in the United States die annually from colorectal adenocarcinomas (Siegel et al., 2018). New tools for precision medicine are necessary to build upon decades of progress in diagnosing and treating colon cancer. Immune Checkpoint inhibition (ICI) therapies, which block interactions between ligands and receptors, are one such innovation that have shown durable anti-tumor response. A combination of both intrinsic and extrinsic tumor features has been shown to correlate with response to checkpoint blockade therapy. Extrinsic factors, such as programmed cell-death ligand 1 (PD-L1) expression in the tumor microenvironment have been shown to correlate with responses to checkpoint blockade therapy (Topalian et al., 2012). More recently, two intrinsic factors related to tumor genetics, microsatellite instability (MSI), and tumor somatic mutation burden (TMB), have been linked to high ICI response rates (Snyder et al., 2014; Le et al., 2015). The high overall response rate (ORR) of solid tumors that are MSI-high (MSI-H) has led to the first tissue agnostic approval for a cancer therapy by the FDA in MSI-H metastatic tumors (Le et al., 2017; U.S. Food and Drug Administration, 2017). However, individual tumors continue to display a range of responses to checkpoint inhibition, highlighting the need for additional research to improve biomarkers and therapeutic approaches.

microRNAs (miRNA) are small, non-coding RNAs that usually function to regulate the expression of a particular gene by depleting the cellular protein contents. This is achieved post-transcriptionally through binding of miRNA to a complementary part of the mRNA transcript for a specific protein. The binding of miRNA to mRNA largely takes place in the 3′ untranslated region and results in either a particular mRNA not being translated or its degradation by the RNA interference effector complex (RISC) (Catalanotto et al., 2016). Because of their importance in many cellular processes, the discovery of miRNAs has led to major advances in understanding and treatment of diseases including pharmocologic approaches. In the first pharmacologic use of miRNAs, Krutzfeldt et al. (2005) showed that a 23-nucleotide RNA molecule, complementary to the miR-122 target, could be delivered to liver tissue ablating endogenous miR-122.

Dysregulated miRNAs are common feature of tumor cells that target oncogenes, tumor suppressor genes, and key immunologic pathways for tumorigenesis (Zhou et al., 2014; Chen et al., 2016; Fang et al., 2018; Vannini et al., 2018). miRNAs have been identified as important aspects of the molecular circuitry underlying cancer—miR-155, for example, has been found to be upregulated in many cancers. Van Roosbroeck et al. (2017) demonstrated that miR-155 directly targets *TP53*, thus functioning as an oncogene. Up till now, there have been several publications concerned with miRNA-based signatures in CRC screening programs. For example, miR-320d is found to be a promising non-invasive diagnostic biomarker that can significantly distinguish the metastatic from non-metastatic CRC patients (Tang et al., 2019). miR-378a-3p were identified as a potential circulating marker to differentiate the CRC patients from healthy subjects (Zanutto et al., 2020). Decreased exosomal miR-139-3p expression may take a role as a novel biomarker for early diagnosis monitoring in CRC patients (Liu et al., 2020). miRNAs have also been found to play an important role in regulating the immune environment. In addition to functioning as an oncogene, miR-155 was found by Lu et al. (2016) to promote M1 polarization along with miR-147-3p, and miR-9-5p. But there are still very few studies focusing on the association of miRNAs with immune therapy through checkpoint inhibitors in CRC.

In addition, the development of therapeutic targets that utilize RNA interference is an active area of pharmacologic research. Our team also sought to further explore the biology of MSI-H tumors and suggest potential companion therapeutics to current checkpoint inhibitors. To do this, we initiated an *in silico* study to look at all three molecular phenotypes indicative of response to ICI therapeutics in the colon and rectal adenocarcinoma (CRC) cohorts from The Cancer Genome Atlas (TCGA) and further characterized changes in both the miRNA and transcriptomes.

## Materials and Methods

### Gathering Data

COAD data from The Cancer Genome Atlas was selected for analysis because many different types of analysis were available for the same patient cohort including: somatic mutation burden, MSI status, mRNA analysis, and miRNA analysis. For our TCGA cohort, miRNA and mRNA expression data were procured from the Broad Firehose (Firehose, 2016). Somatic mutation calls were obtained from the Genomic Data Commons for all CRC patients in TCGA (Grossman et al., 2016).

### Obtaining Tumor Features

**FIGURE 1:**
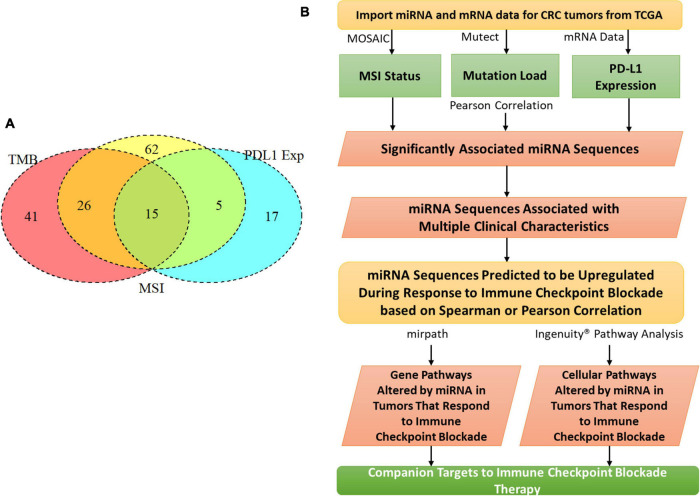
MiRNAs correlated with clinical features related to immunotherapy. **(A)** Tumor mutation burden, programmed death ligand 1 expression, CD8 fraction, and microsatellite instability were analyzed for a cohort of 549 colorectal cancer patients in The Cancer Genome Atlas. 15 miRNAs were identified that correlated with all 3 clinical features. **(B)** The whole analysis pipeline of the whole project.

We chose tumor pathologies previously associated with response to checkpoint blockade immunotherapy for assessment in our CRC patient cohort from TCGA (Figure 1B). To compare and contrast miRNA expression between these tumor features, we also compared tumor phenotypes where one would expect a great deal of overlap, for example, MSI and TMB. MSI was assessed with the MicrOSAtellite Instability Classifier (MOSAIC) from Hause et al. (2016) to predict MSI status based on Whole Exome Sequencing (WES) data. The proportion of unstable microsatellite loci across the exome was correlated with the expression of miRNA. TMB was assessed using Mutect2 and a 5% cutoff for allele frequency (Cibulskis et al., 2013). Expression of PD-L1 was assessed by quantifying gene expression—FPKM values from TCGA were used for this.

### Statistical Analysis

To assess whether each tumor feature was correlated with the presence of a particular miRNA, a Pearson correlation coefficient was used. miRNAs were individually assessed for correlation with each tumor feature. Once correlations were assessed for the different tumor features, miRNAs were pooled to look for miRNAs that were correlated with all 3 tumor features.

### Immune Cell Deconvolution

In order to alleviate bias from any one algorithm, three separate tools were used to assess immune cell populations: xCell, TIMER, and CIBERSORT. CIBERSORT reports the fraction of 22 different immune cell lineages that are present in a particular RNA-Seq sample (Chen et al., 2018). xCell, similar to CIBESORT, is a gene signature-based method used to infer 64 immune and stromal cell types (Aran et al., 2017). The Tumor Immune Estimation Resource (TIMER) allows the calculation of six tumor-infiltrating immune subsets from gene expression data (Li et al., 2017). *T*-tests were used for each algorithm to determine whether the fraction of immune cells differed between phenotypic classifications of tumors. Once the group of miRNAs was determined to influence macrophage polarization in aggregate, each miRNA was individually assessed to determine whether it was correlated with macrophage polarization.

### Pathway Analysis

mirPath (v3), a tool for predicting gene targets of miRNA sequences, was used to analyze which pathways the selected group of miRNA would preferentially (Vlachos et al., 2015). Once miRNA’s were identified that correlated with macrophage polarization, these miRNAs were analyzed with mirPath to see which genes and pathways were targeted. TargetScan was queried using a conservation score of 0.1 to find genes and pathways intersected by miR-22, miR-155, or miR-146b (Karagkouni et al., 2018). Cancer-related genes and pathways were selected from those targeted by these miRNAs.

## Results

### Patient Cohort

The CRC patient cohort (*n* = 549) from TCGA was made up of 406 colon adenocarcinoma (COAD) patients and 143 rectal adenocarcinoma (READ) patients (Supplementary Table 1). More detailed information is shown in Supplementary Table 2. Typical immunotherapy recipients have late-stage cancers—we looked at stage in order to ensure a patient population representative of current immunotherapy recipients. We found that 14% of the total CRC patient cohort was advanced stage (IV). CRC patients were MSI-H at a rate of 18% in our CRC cohort, consistent with the literature. We chose three clinical features previously found to influence response to immunotherapy: MSI, tumor mutation burden (TMB), and PD-L1 expression. By aggregating these features, we aimed to predict an immunogenic subset of tumors from TCGA.

### miRNAs Associated With Clinical Features Related to Immunotherapy

To characterize the relationship between miRNAs and the clinical features analyzed, a Pearson correlation was chosen. We measured linear correlations between each clinical variable and miRNA expression. Our Pearson correlation analysis resulted in 41 miRNAs significantly correlated with mutation burden, 62 miRNAs significantly correlated with MSI, and 17 miRNAs significantly correlated with PD-L1 expression. Of these three lists, 15 miRNA were overlapped and 12 of them were consistently positively correlated with the 3 tumor features and three of them were negatively correlated with the three tumor features (Figure 1A). 15 of these miRNAs were used for further analysis because they were correlated with all three tumor features (Table 1). To further characterize the 15 miRNA that were correlated with our clinical features, we conducted pathway analysis revealing 2 immune-related pathways for further exploration: Colorectal cancer and TGF-β.

**TABLE 1:** miRNAs associated with 3 tumor phenotypes.

**miRNA**	Association MMR- tumor features*	**Associated with survival? (Z score)**	**Associated with macrophage polarization**
*let-7i*	Up	No	No
*mir-1266*	Up	No	No
*mir-132*	Up	No	No
*mir-146b*	Up	No	Yes
*mir-155*	Up	No	Yes
*mir-212*	Up	No	No
*mir-22*	Up	No	Yes
*mir-223*	Up	No	No
*mir-511 (3p/5p)*	Up	No	No
*mir-625*	Up	No	No
*mir-629*	Up	No	No
*mir-335*	Down	No	No
*mir-552*	Down	No	No
*mir-92a*	Down	No	No

*MiRNAs found to be associated with 3 tumor phenotypes through the analysis described in Figure 1B. 15 miRNAs were found to have a common association with all 3 tumor phenotypes using Pearson’s correlation. Whether the association was positive (Yes) or negative (No) was determined from the correlation coefficient, association with survival was determined using the R survival package, and association with macrophage polarization was determined using CIBERSORT and Pearson’s correlation.

### Association of Clinical Feature Related to Immunotherapy With Immune Cell Types

**FIGURE 2:**
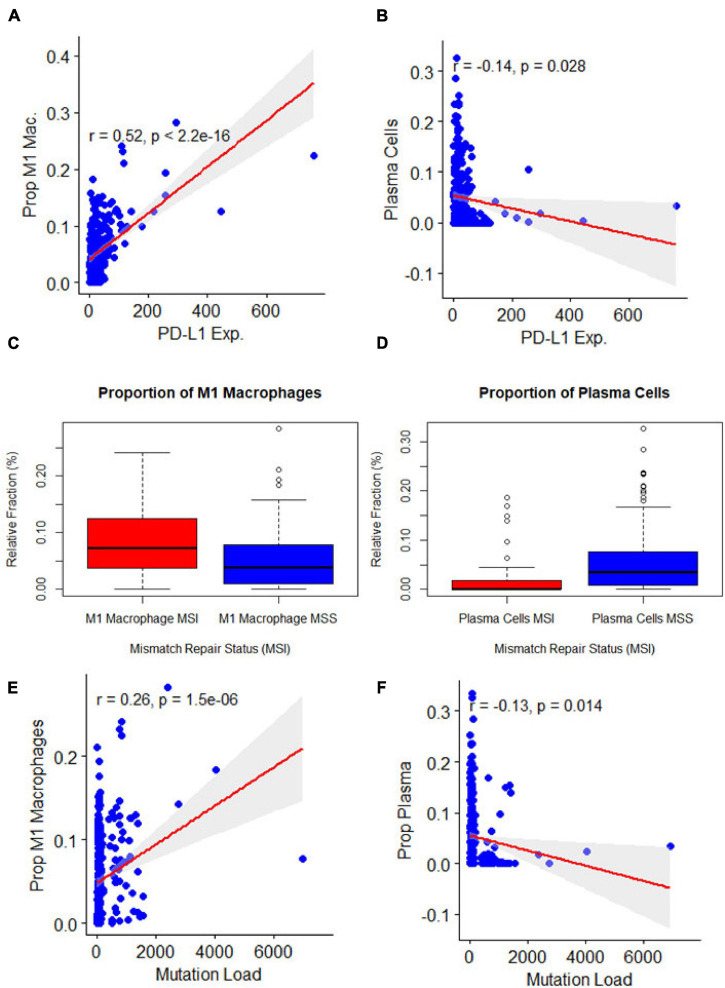
Association of clinical features related to immunotherapy with immune cell types **(A,B)**. Association of microsatellite instability status with: M1 macrophage polarization (*p* = 0.00) and plasma cells (*p* = 0.00). **(C,D)** Association of programmed death-ligand 1 expression with: M1 macrophage polarization (*p* = 0) and plasma cells (*p* = 0.03), y axis represented the immune cell deconvolution results as fraction relative to the immune-cell content: M1 macrophage and plasma cells. **(E,F)**: Association of mutation burden with: M1 macrophage polarization (*p* = 0.00) and plasma cells (*p* = 0.01).

**FIGURE 3:**
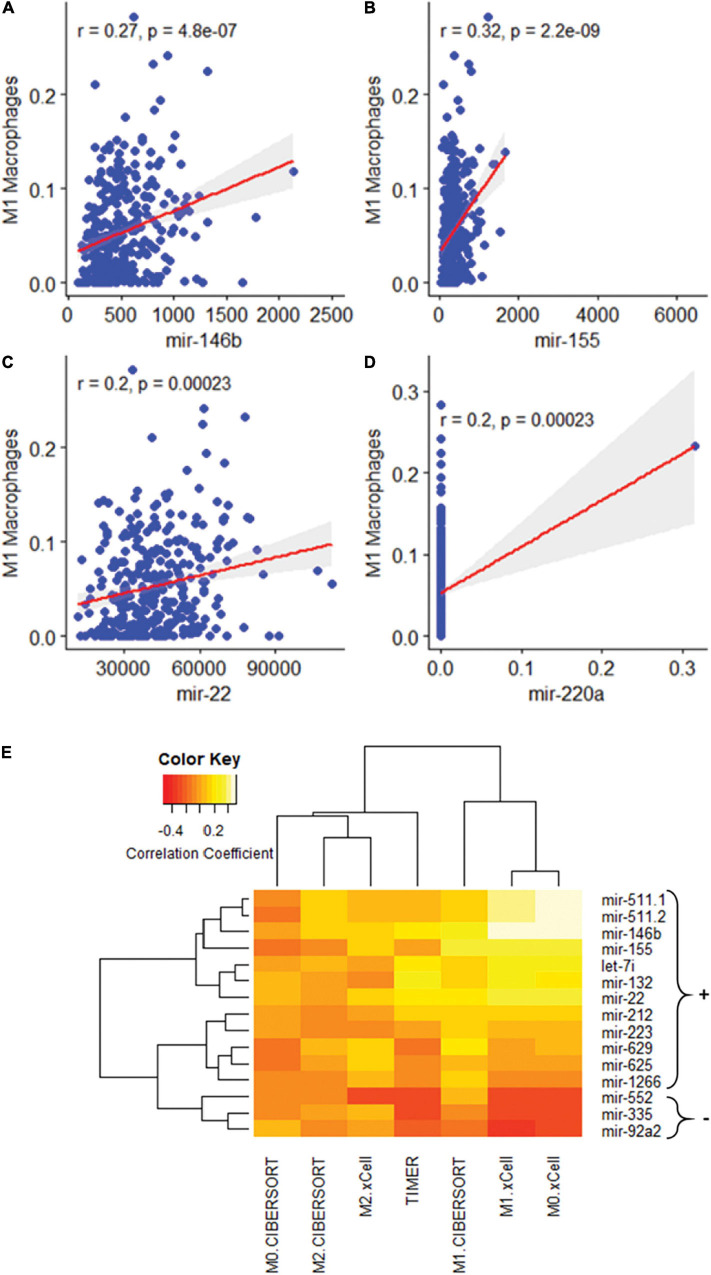
Association of M1 macrophage polarization with miRNAs. **(A–D)** Association of M1 macrophage polarization with: mir-146b (*p* = 0.00), mir-155 (*p* = 0.00), and mir-22 (*p* = 0.00). mir-220a was excluded as the correlation was the result of a single outlier. **(E)** A heatmap of 15 miRNA sequences correlated with macrophage polarization.

MSI, TMB, and PD-L1 expression were all separately assessed for Pearson correlations with the proportions of different immune cell types as reported by three separate immune cell deconvolution algorithms. Out of many cell types, only the proportions of plasma cells and M1 macrophages were significantly correlated with all three tumor features. The proportion of M1 macrophages was highly positively correlated with PD-L1 expression (Figure 2A, *p* < 0.001), MSI (Figure 2C, *p* = 0.001), and TMB (Figure 2E, *P* < 0.001). However, the proportion of plasma cells was negatively correlated with PD-L1 expression (Figure 2B, *p* < 0.001), MSI (Figure 2D, *p* = 0.001), and TMB (Figure 2F, *P* < 0.001) To further characterize the relationship between the proportion of M1 macrophages and the three tumors analyzed, we looked at correlations between M1 macrophage proportion and the expression of individual miRNAs. Among the 15 miRNAs, we found three miRNAs were significantly correlated with both the three clinical characteristics and M1 macrophage polarization: miR-22, miR-146b, and miR-155 (Figure 3). One miRNA, miR-220a, was excluded from further analysis because the correlation was based entirely on a single outlier.

### Pathway Analysis

To characterize the crucial pathways for modulating the tumor immune environment, we predicted pathways that would be influenced by the three miRNAs related to both macrophage polarization and three tumor features. Unsurprisingly, these three miRNAs were predicted to influence the expression of genes in key immune and cancer-related pathways (Table 2). As a group, miR-155 and miR-22 were predicted to strongly influence pathways related to Colorectal Cancer (*p* = 0.0001) and TGF-β signaling (*p* = 0.008). Out of 21 genes predicted to be influenced by these miRNAs, three genes were shared between pathways related to COAD and TGF-β signaling: SMAD2, SMAD4, and TGFBR2.

**TABLE 2:** Genes targeted by miRNAs interact with in the TGF-β pathway and colorectal cancer pathway.

Gene*	**Ensembl ID**	**TGF-β**	**CRC**	**hsa-miR-155-5p**	**hsa-miR-22-3p**
SMAD2	ENSG00000175387	Yes	Yes	Yes	
ACVR1B	ENSG00000135503	Yes	No		Yes
SKP1	ENSG00000113558	Yes	No		Yes
ACVR2B	ENSG00000114739	Yes	No	Yes	Yes
SMAD4	ENSG00000141646	Yes	Yes		Yes
ZFYVE9	ENSG00000157077	Yes	No		Yes
ACVR2A	ENSG00000121989	Yes	No	Yes	
SP1	ENSG00000185591	Yes	No		Yes
EP300	ENSG00000100393	Yes	No		Yes
TGFBR2	ENSG00000163513	Yes	Yes	Yes	
FOS	ENSG00000170345	No	Yes	Yes	
GSK3B	ENSG00000082701	No	Yes	Yes	
PIK3CB	ENSG00000051382	No	Yes		Yes
KRAS	ENSG00000133703	No	Yes	Yes	
TP53	ENSG00000141510	No	Yes		Yes
PIK3CD	ENSG00000171608	No	Yes		Yes
CCND1	ENSG00000110092	No	Yes	Yes	
PIK3R1	ENSG00000145675	No	Yes	Yes	
AKT3	ENSG00000117020	No	Yes		Yes
PIK3CA	ENSG00000121879	No	Yes	Yes	
MAPK10	ENSG00000109339	No	Yes		Yes

*MiRNA associated with microsatellite instability status, somatic tumor mutation burden, PD-L1 expression, M1 macrophage polarization that interact with the TGF-β signaling pathway (p = 0.008) and CRC pathways (p = 0.0001). Most of these genes interact with 2 miRNA sequences: hsa-miR-155-5p (p = 0.004) and hsa-miR-22-3p (p = 0.038). miRNA associations with genes were predicted by TargetScan (Conservation Score = 0.1). Results for TGF-β were merged by pathway union and results for CRC were merged by gene union.

## Discussion

The aim of this study was to explore new targets for checkpoint blockade immunotherapy by exploring the unique biology of tumors known to respond to these drugs. Three features common to such tumors including high mutation burden, MSI, and PD-L1 expression were added into analysis. These features had 15 miRNAs in common, however, none of the 15 miRNAs predicted survival. M1 macrophage were found correlated with all three features through Pearson Correlation analysis. As a group, these 15 miRNAs predicted macrophage polarization. Individually assessing each of the 15 miRNAs for a correlation with macrophage polarization revealed three miRNAs that were strongly correlated with macrophage polarization: miRNA-146b, miRNA-155, and miRNA-22. Subsequent pathway analysis revealed these three miRNAs as important components of the TGF-β and Colorectal Cancer pathways.

**FIGURE 4:**
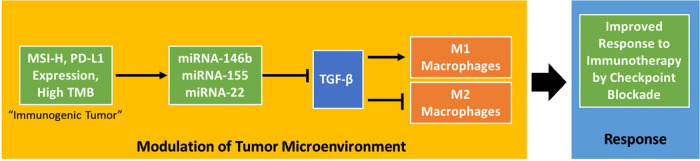
A potential mechanism by which MSI status, PD-L1 expression, and tumor mutation burden influence the tumor microenvironment. The proposed mechanism shows that these tumor phenotypes influence the tumor microenvironment in a TGF-β-dependent way to improve response to checkpoint blockade immunotherapy.

In this study, we searched all possible datasets from TCGA and GEO, TCGA is the only dataset that both contains miRNA and transcriptome, and our study analyzed TCGA data as controlling for bias using a randomly selected testing/training dataset. MicroRNA has been regarded as important promising molecular biomarkers in several tumor types (Zhang et al., 2013; Chou et al., 2017). TGF-β has been identified as inhibiting the expansion and function of many components of the immune system (Batlle and Massague, 2019). A recent pair of papers has shown TGF-β to be an important modulator of the tumor microenvironment (Mariathasan et al., 2018; Tauriello et al., 2018). These experiments identify TGF-β signaling as an important aspect of response to PD-1-PD-L1 immunotherapy, connecting it to lower proportions of T cells in the tumor and poorer responses. This research supports the discovery of miRNAs targeting TGF-β in immunogenic tumors. TGF-β has also been shown to modulate the proportion of macrophages in the tumor microenvironment, promoting their polarization to an M2-like phenotype (Gong et al., 2012). Both ideas support a key role for suppressing TGF-β in immunogenic tumors that respond to checkpoint blockade therapy. Our research further characterizes this interaction by suggesting dysregulation of miRNA in immunogenic tumors as part of the biological system enabling responses to checkpoint blockade drugs (Figure 4).

Although, we didn’t have validation of miRNA panel, TGF-β has been widely identified in many biological experiments which can be solid support evidence for the hypothesis we presented in this research. For example, miR-146b has been found to inhibit TGF-β by binding to the 3′ untranslated region (UTR) of SMAD4, an important member of the signaling pathway. Increased SMAD 4 levels and decreased cellular proliferation was observed by Geraldo et al. (2012) in human papillary carcinoma cells. Another study found the overexpression of SMAD4 in BCPAP cells, which is a validated target of miR-146b-5p and key protein in the TGF-β signaling pathway, significantly decreased migration and invasion to a degree very similar to that observed with the antagomir-146b-5p (Lima et al., 2016). miR-155 is one of the most extensively studied miRNAs and was the first miRNA shown to be oncogenic. An extensive body of research has established an important role for miR-155 throughout cellular process related to human cancer (Costinean et al., 2006; Volinia et al., 2006). Geraldo et al. (2012) showed that miR-22 is significantly downregulated in TGF-β treated HT-29, a commonly used human colorectal cancer cell line (Cai et al., 2013).

In this study, we identified three miRNAs common in three immunotherapy-related clinical characteristics as well as M1 macrophage polarization, and function prediction of miRNAs showed SMAD2, SMAD4, and TGFBR2 were in common from COAD and TGF-β signaling pathways. miR-155 and miR-22 could influence pathways related to Colorectal Cancer and TGF-β signaling. Previous studies have already proved the regulation function of SMAD2, SMAD4, and TGFBR2 in cancers (Matsuzaki et al., 2009; Zhu et al., 2020), and these genes were also found related with miRNAs that strongly correlated with tumor features, indicating the potential function and clinical utility in immunotherapy.

## Conclusion

Our comprehensive, integrated analysis of three miRNAs in colorectal cancer revealed a crucial component of TGF-β that modulate tumor immune environment and significantly correlated with macrophage polarization. The work highlights the important clinical implications of miRNAs functions in checkpoint blockade immunotherapy and helps develop potential therapeutical strategies for CRC patients.

## Data Availability Statement

The datasets presented in this study can be found in online repositories. The names of the repository/repositories and accession number(s) can be found in the article/Supplementary Material.

## Ethics Statement

Existing data was used with permission and requested from dbGap and the Genomic Data Commons. No human subjects’ data was collected.

## Author Contributions

BB, ZG, and YD conducted the study and prepared the manuscript. BB, ZG, PF, and YD revised the manuscript. VK and MM provided statistical and informatics support and helped prepare the figures. MS and MF provided biological expertise in analyzing miRNA data. All authors have read and agreed to the published version of the manuscript.

## Conflict of Interest

The authors declare that the research was conducted in the absence of any commercial or financial relationships that could be construed as a potential conflict of interest.

## Publisher’s Note

All claims expressed in this article are solely those of the authors and do not necessarily represent those of their affiliated organizations, or those of the publisher, the editors and the reviewers. Any product that may be evaluated in this article, or claim that may be made by its manufacturer, is not guaranteed or endorsed by the publisher.

## Abbreviations

ORR: Overall Response Rate
MSI: Microsatellite Instability
MSS: Microsatellite Stable
COAD: Colon adenocarcinoma
READ: Rectal Adenocarcinoma
MOSAIC: MicrOSAtellite Instability Classifier
TCGA: The Cancer Genome Atlas
TGF- β: Transforming growth factor beta.
